# Ultrahigh field MRI in clinical neuroimmunology: a potential contribution to improved diagnostics and personalised disease management

**DOI:** 10.1186/s13167-015-0038-y

**Published:** 2015-08-27

**Authors:** Tim Sinnecker, Joseph Kuchling, Petr Dusek, Jan Dörr, Thoralf Niendorf, Friedemann Paul, Jens Wuerfel

**Affiliations:** NeuroCure Clinical Research Center (NCRC), Charité - Universitaetsmedizin Berlin, Charitéplatz 1, 10117 Berlin, Germany; Department of Neurology, Asklepios Fachklinikum Teupitz, Buchholzer Str. 21, 15755 Teupitz, Germany; Institute of Neuroradiology, Universitaetsmedizin Goettingen, Robert-Koch-Straße 40, 37075 Goettingen, Germany; Department of Neurology and Center of Clinical Neuroscience, Charles University in Prague, 1st Faculty of Medicine and General University Hospital in Prague, Kateřinská 30, 128 21 Praha 2, Czech Republic; Clinical and Experimental Multiple Sclerosis Research Center, Department of Neurology, Charité Universitaetsmedizin Berlin, Charitéplatz 1, 10117 Berlin, Germany; Berlin Ultrahigh Field Facility, Max Delbrueck Center for Molecular Medicine, Robert-Roessle-Strasse 10, 13125 Berlin, Germany; Experimental and Clinical Research Center, Charité - Universitaetsmedizin Berlin and Max Delbrueck Center for Molecular Medicine, Robert-Roessle-Strasse 10, 13125 Berlin, Germany; Department of Neurology, Charité - Universitaetsmedizin Berlin, Charitéplatz 1, 10117 Berlin, Germany; Medical Image Analysis Center, Mittlere Strasse 83, CH-4031 Basel, Switzerland

**Keywords:** 7 Tesla, Ultrahigh field MRI, Multiple sclerosis, Neuromyelitis optica, Susac syndrome, Neuroimmunology, Central vein sign, Cortical lesions, Predictive, Preventive and Personalised Medicine

## Abstract

Conventional magnetic resonance imaging (MRI) at 1.5 Tesla (T) is limited by modest spatial resolution and signal-to-noise ratio (SNR), impeding the identification and classification of inflammatory central nervous system changes in current clinical practice. Gaining from enhanced susceptibility effects and improved SNR, ultrahigh field MRI at 7 T depicts inflammatory brain lesions in great detail. This review summarises recent reports on 7 T MRI in neuroinflammatory diseases and addresses the question as to whether ultrahigh field MRI may eventually improve clinical decision-making and personalised disease management.

## Review

### Introduction

Magnetic resonance imaging (MRI) revolutionised clinical neuroimmunology since brain MRI depicted multiple sclerosis (MS) lesions already in early technical developmental stages at 0.1 Tesla (T) [1]. During the past decade, MRI became a crucial tool to diagnose and monitor inflammatory central nervous system (CNS) alterations [2]. Nonetheless, today’s physicians are faced with a key issue in clinical neurology: many distinct CNS diseases are characterised by nearly identically appearing white matter changes and brain lesions that are often unspecific in appearance, limiting the diagnostic value of conventional MRI.

Ultrahigh field (UHF) MRI at 7 T benefits from increased signal-to-noise ratio (SNR) and enhanced spatial resolution as good as 100 μm [3]. Future studies will show whether these 7 T MRI advantages indeed improve diagnosis and our understanding of the underlying pathophysiology in inflammatory CNS diseases. Following the recommendations of the "EPMA White Paper" [4], this review summarises technical opportunities, challenges, and findings of recent clinical 7 T MRI studies on multiple sclerosis, neuromyelitis optica, and Susac syndrome.

### Technical improvements and limitations

**Fig. 1:**
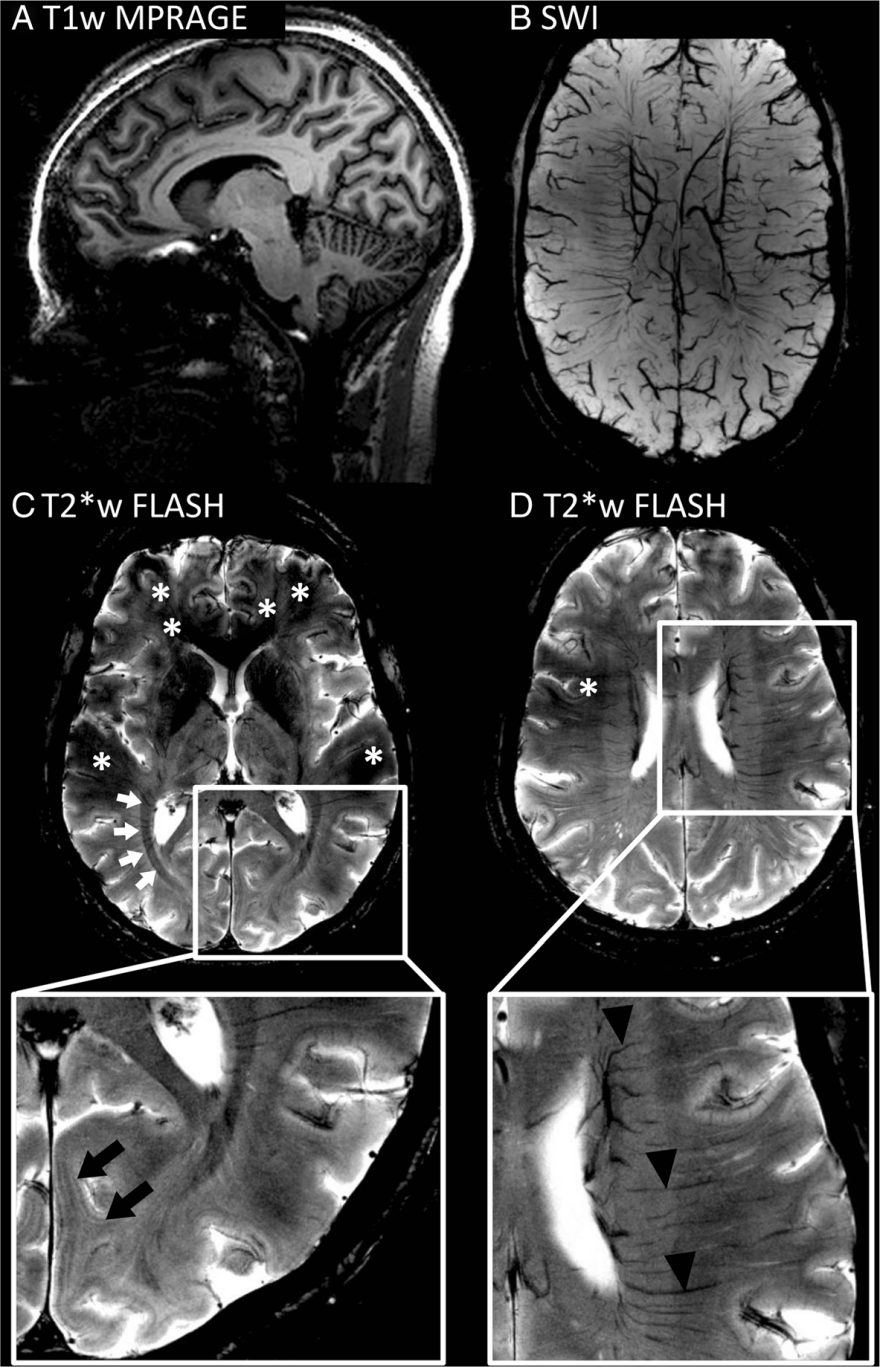
Brain structures visualised on 7 Tesla MRI images. **a** 7 T T1w MPRAGE provides high-resolution anatomical imaging with excellent gray to white matter contrast. **b** 7 T SWI depicts very small brain veins. **c**, **d** 7 T T2*w FLASH with a resolution of 0.2 mm × 0.2 mm × 2 mm delineates strongly myelinated structures such as the optic radiation (*white arrows*) or the stripe of Gennari (*black arrows*, zoom). In addition, very small veins are visualised in the periventricular white matter (*black arrowheads*, zoom). Nevertheless, the image quality of 7 T gradient echo images is sometimes reduced due to inhomogeneities or artifacts (*asterisks*)

SNR is a key factor in MRI and the currency spent for diagnostic accuracy. Although the level of background noise increases proportionally with magnetic field strengths, the magnitude of the MR signal even gains by square [5], causing the SNR to increase nearly linearly with the magnetic field strength [6]. Consequently, increased SNR at 7 T can be used to acquire MR images of very high spatial resolution, e.g., up to 0.08 mm^3^ (Fig. 1). Furthermore, UHF MRI benefits (and sometimes suffers) from increased susceptibility effects that are caused by, e.g., paramagnetic or ferromagnetic substances such as iron species (mostly ferritin and haemosiderin) and deoxyhaemoglobin. These microscopic disturbances of the magnetic field on cellular and tissue levels cause a focal signal loss resulting from dephasing spins during gradient echo image acquisitions and a positive (paramagnetic) phase shift of the MR signal. Hence, not only very small brain structures containing paramagnetic substances such as veins but also highly aligned or densely myelinated structures such as the optic radiation or even the small line of Gennari that is part of the primary visual cortex may be visualised in 7 T T2* weighted (T2*w) images (Fig. 1). Furthermore, deep brain stem structures such as nerve roots, or pons fibers [7], and the habenula [8] can now be visualised in great detail.

However, there are still few practical and technical considerations to be made when applying UHF MRI: Some patients may be excluded from an examination at 7 T due to an increased number of contraindications at UHF as compared to lower field strengths, such as tattoos, dental implants, metallic intrauterine devices, stents, surgical clips, and piercings. These may also include otherwise "MRI-safe" implants such as pacemakers or orthopaedic replacements.

Furthermore, there are technical challenges that deserve attention: Increased magnetic field inhomogeneity may impact post-processing procedures despite excellent gray to white matter contrast. Radiofrequency (RF) power deposition constitutes another practical challenge since it scales superlinearily with the magnetic field strength. Local RF coils that offer improved transmission efficiency versus large volume coils can be instrumental to offset this challenge [9–11].

When considering these constraints, UHF MRI is believed to be safe and it is well tolerated by the vast majority of patients [12, 13]. Nonetheless, temporary adverse events were reported during 7 T at higher frequency compared to 1.5 T MRI [14]. In addition, 5 % of all subjects or patients reported vertigo during UHF MR exams [14]. During scan with magnetic field gradients being rapidly switched, visual disturbances or temporary muscle contractions may occur [15–17]. Deteriorating vital signs or long-term effects have—to the best of our knowledge—not been described during or after 7 T MRI investigations [13, 18, 19], but the relevance of preliminary in vitro studies on potential deoxyribonucleic acid (DNA) damage caused by a static magnetic field of 1.5 T or by rapidly changing magnetic fields is still subject to discussion [20, 21]. A recent analysis of DNA double-strand breaks (DSB) in human peripheral blood mononuclear cells after exposure to 7 T did not show a significant increase in DSB levels compared to the unexposed control group [16].

### Multiple sclerosis

Multiple sclerosis is an inflammatory and neurodegenerative autoimmune CNS disorder affecting white as well as gray matter of the brain and spinal cord [22–24]. The disease is characterised by a wide range of symptoms and a large heterogeneity in clinical presentation. Besides neurological impairment in visual, pyramidal, cerebellar, sensory, and vegetative functional systems, more global symptoms of CNS dysfunction such as fatigue and cognitive dysfunction may occur that negatively impact patients’ quality of life [23, 25–30]. MRI and more recently optical coherence tomography (OCT) have emerged as valuable imaging tools for contributing to diagnosis, differential diagnosis, and disease monitoring [31–39]. These imaging techniques have shown that beyond focal lesions, diffuse and widespread tissue damage occurs in both the gray and the white matter already in early disease stages [40–43] and more pronounced in progressive disease [44]. However, diagnosis and treatment decisions in clinical routine are still widely based on the detection of focal cerebral white matter lesions hyperintense on T2 weighted (T2w) or fluid attenuated inversion recovery (FLAIR) images. An accurate diagnosis of MS remains challenging given the insufficient specificity of focal white matter lesions [45, 46]. In this regard, UHF MRI improves both the detection and morphological description of MS lesions and may thus be used in the future to distinguish MS from lesions of other origins and to improve our understanding of the disease. This is of high clinical relevance as the broadening MS treatment landscape will pave the way for an individualised and tailored MS therapy [47]. However, with the increasing number of available efficacious immunosuppressive and immunomodulatory drugs for MS, a correct and timely diagnosis is a prerequisite for personalised medicine that weighs benefits and risks of these drugs in every individual patient [24, 48–54].

#### Cortical gray matter lesions

The detection of cortical lesions is greatly improved by 7 T MRI [55]. Gray matter pathology accumulates during disease progression and may affect major areas of the cortex in long-standing multiple sclerosis [56–58]. Recent studies revealed that cortical lesions are associated with disease progression, disability, and cognitive dysfunction [59–61]. In conventional MRI, the vast majority of cortical lesions remain undetected even when applying double inversion recovery (DIR) techniques at 1.5 T [62, 63]. UHF MRI at 7 T improves the detection of cortical lesions and depicts up to 48 % of all cortical lesions revealed by ex vivo immunohistochemical staining for myelin [64]. These results were confirmed by several in vivo studies. Magnetisation transfer imaging at 7 T was reported to detect roughly 25 % more cortical lesions than 3 T DIR in a recent study [65]. Furthermore, 7 T 3D FLAIR is highly sensitive in detecting cortical lesions and detects 89 % more lesions than 7 T 3D DIR [66]. A multi-contrast 3 T versus 7 T comparative study reported 7 T MRI to detect up to 238 % more cortical lesions than 3 T [67]. In addition, it was shown that 7 T T1 weighted magnetisation prepared rapid acquisition gradient echo (MPRAGE) imaging increases the detection rate of cortical lesions by twofold in comparison to 1.5 T MPRAGE [65, 68].

**Fig. 2:**
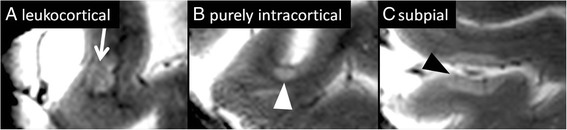
Cortical gray matter lesions in multiple sclerosis. Cortical gray matter lesions can be differentiated into distinct lesion subtypes on 7 T T2*w images. Leukocortical (type I) lesions (**a**) cross the border (*white arrow*) between the white and the gray matter. Purely intracortical (type II) lesions (**b**) are commonly small and centered on a small blood vessel (*white arrowhead*). Finally, subpial (type III/IV) lesions (**c**, *black arrowhead*) grow from the subpial cortical area into the cortex. *The purely intracortical (type II) lesion depicted in this figure has previously been published in: “Ultrahigh field MRI in context of neurological diseases.” Kuchling J, Sinnecker T, Bozin I, Dörr J, Madai VI, Sobesky J, Niendorf T, Paul F, Wuerfel J. Nervenarzt. 2014;85(4):445–58. doi:* 10.1007/s00115-013-3967-5. [3]

Owing to the high spatial resolution at UHF, cortical lesions are much easier to be differentiated from subcortical lesions—or artifacts—at 7 T compared to 3 T MRI [55]. Accordingly, an excellent inter-rater-reliability of 7 T (*k* = 0.97) was reported in contrast to 3 T DIR (*k* = 0.12) in detecting cortical lesions [69]. Most importantly, UHF MRI can differentiate the various cortical lesion subtypes as defined by histology [70], including leukocortical (type I) lesions, purely intracortical (type II) lesions, and subpial (type III/IV) lesions (Fig. 2) [71–74]. The latter were found to be very specific for MS in a histopathological study [75]. Interestingly, 7 T T2*w fast low angle shot (FLASH) is superior over 3 T DIR in detecting subpial (type III/IV) lesions [69]. Accordingly, a recent study using the T2* mapping technique at 7 T revealed subpial T2* relaxation time changes in large cortical areas in long-standing MS [76]. In addition, thalamic gray matter lesions visualised on 7 T MRI images correlate with disability and are more often detectable in progressive MS [77].

In sum, there is increasing evidence that 7 T MRI detects significantly more (subpial) cortical lesions than 3 T, but the detection of some type III lesions still remains challenging [69].

#### Improved depiction of white matter lesions

Persisting T1 weighted (T1w) hypointense lesions—namely black holes—contribute to disability in MS in addition to cortical lesions [78, 79]. At UHF strength—and in contrast to conventional MRI at 1.5 T—virtually, every T2w hyperintense lesion is visible as a distinct hypointense plaque on 7 T T1w MPRAGE images as shown by our group and others [68, 80]. Contrarily, 1.5 T T1w MPRAGE delineated only 68 to 78 % of T2w lesions in the same study [68]. Moreover, 7 T T1w MPRAGE is even more sensitive in detecting MS lesions than 1.5 T T2w (728 versus 545 lesions) [68] or 3 T FLAIR imaging (1043 versus 812 lesions) [80].

In contrast to these improvements, 7 T T2w or FLAIR does not depict a significantly higher lesion count compared to 3 T T2w MRI [67, 81].

**Fig. 3:**
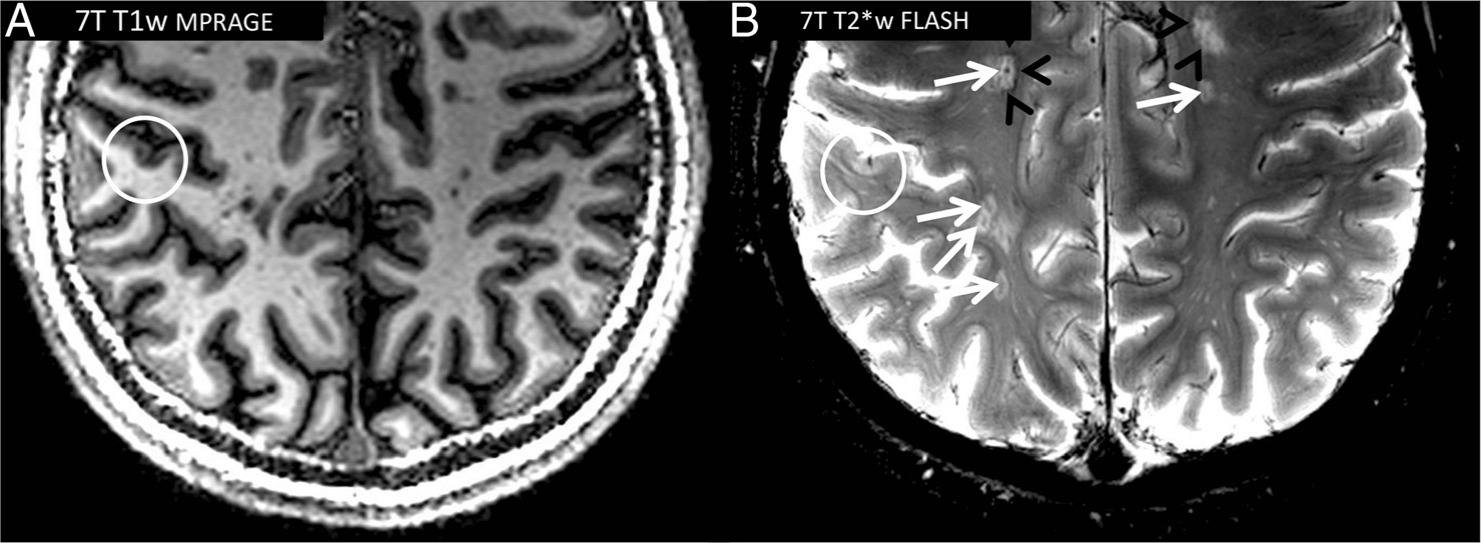
Exemplary multiple sclerosis lesions. 7 T T1w MPRAGE (**a**, spatial resolution 1.0 mm × 1.0 mm × 1.0 mm) and 7 T T2*w FLASH images (spatial resolution 0.5 mm × 0.5 mm × 2.0 mm) are displayed. 7 T T2*w FLASH delineates various multiple sclerosis white matter lesions that are centered on a small venous vessel (*white arrows*). In addition, a hypointense rim can be depicted at the edge of a proportion of white matter lesions (*black arrowheads*). A subpial (Type III/IV) lesion is visible in the right hemisphere (*circle*) as a T2*w hyperintensity (**b**) and a corresponding T1w hypointensity (**a**) within the cortical gray matter

In conclusion, 7 T T1w MPRAGE is highly sensitive in detecting MS white matter lesion damage (Fig. 3a), but the T2w lesion count is not substantially increased at 7 T compared to 3 T MRI. The true advantage of 7 T T2w imaging is the visualisation of very small morphological lesion details as described in the following paragraph.

#### White matter lesion morphology

Gaining from increased susceptibility effects and spatial resolution, T2*w imaging at 7 T delineates distinct morphological features of MS lesions. Most importantly, a very small vein can be displayed within the center of the MS lesion on T2*w images, and the lesion often follows the course of the vessel (Fig. 3b) [71, 73, 74, 81, 82]. This feature is not only detectable in relapsing-remitting MS but also observable in primary progressive MS [83]. In addition, a proportion of MS lesions is characterised by a T2*w hypointense rim surrounding the lesion (Fig. 3b) [71, 73, 74]. A comparative 7 T and histopathological study found that these rims correspond to iron-rich CD68-positive cells of the macrophage lineage [73]. Hence, a positive rather thick rim-like phase shift is detectable around these lesions at 7 T [84]. Contrarily, rather thin rim-like phase shifts around MS lesions without major T2*w hypointensity in these areas were associated with blood-brain barrier breakdown and inflammatory activity [84]. In general, MRI phase imaging can provide additional information on the tissue microstructure that is not encoded in the magnitude of the MR signal. Thus, MRI phase imaging at 7 T depicts white matter lesions prior to conventional T2w imaging as revealed by a case series [85]. Finally, susceptibility changes indicative of iron deposition within the center of a proportion of MS lesions can be found even in the earliest MS disease stages [86]. The origin of these iron deposits, however, still remains unclear and highly speculative. Leakages of haemoglobin through a leaky blood vessel or dying iron-rich oligodendrocytes releasing iron into the extracellular matrix are only two hypotheses among many others [87–91].

#### Differential diagnosis by 7 T MRI

The detailed description of the lesion morphology facilitates the distinction of MS lesions versus brain lesions of other origin [92–95]. A first study on 28 MS patients and 17 subjects with non-symptomatic lesions presumably caused by small vessel disease found that the “central vein sign” differentiates MS patients from these controls by using a central vein cutoff of 40 % [94]. The same cutoff was reported to be beneficial in predicting MS conversion of clinically isolated syndrome (CIS) patients [96]. In detail, each of 13 CIS patients with a positive central vein sign (>40 %) at baseline included in a prospective study developed MS, and all CIS patients (*n* = 9) with a negative central vein sign (<40 %) at baseline were ultimately diagnosed as not having MS [96]. The median follow-up time in this study was 26 months (range, 4–37 months) [96]. Although these initial results must be confirmed in a larger dataset with longer follow-up, this study illustrates the potential predictive capability of 7 T MRI.

#### Venous abnormalities in MS

The controversy on cerebrospinal venous insufficiency in MS [97–100] revitalised a discussion on vascular abnormalities within MS lesions that were first described by Dawson et al. in early 1916 [101]. Today, 7 T T2*w imaging can depict very small brain veins in vivo (Fig. 1) [71, 74, 82, 102]. The venous density is reduced in MS compared to healthy controls presumably as a consequence of hypometabolism, gliosis, and vascular damage [103]. This reduction in (periventricular) venous density is already detectable in the earliest MS disease stages and patients with CIS [103]. Furthermore, shrinkage of intra-lesional compared to extra-lesional veins was reported recently [104]. Although the degree of intra-lesional venous shrinkage was smaller in another study [102], intra-lesional venous shrinking is a potential in vivo imaging marker of inflammation since it is hypothesised to be the consequence of thickened vein walls caused by inflammation leading to obstruction and reduced blood flow [105].

#### Structural damage and atrophy in MS

High-resolution 7 T T2*w imaging visualises strongly myelinated aligned structures such as the optic radiation (OR, Fig. 1). Furthermore, very small lesions can be displayed within the OR on 7 T images [106]. The lesion volume affecting the optic radiation was reported to be associated with OR atrophy and retinal thinning as revealed by OCT [106]. This association between OR damage and retinal atrophy may reflect retrograde transsynaptic degeneration, but independent mechanisms may play a role, too.

Quantifying the total volume of brain tissue and volumes of gray or white matter is impeded at 7 T by the local field inhomogeneity. This limitation may be overcome by a T1w MPRAGE sequence with two inversion pulses, e.g., MPRAGE with multiple echoes (MP2RAGE), a technique recently recommended for generating a homogenised T1w image free of proton density or T2w contrast [107]. Indeed, the MP2RAGE approach yielded sufficient cortical surface reconstructions [108] and voxel-based morphometry (VBM) analyses estimating gray matter volume can be of good quality regarding superior cortical areas [109, 110].

### Neuromyelitis optica

**Fig. 4:**
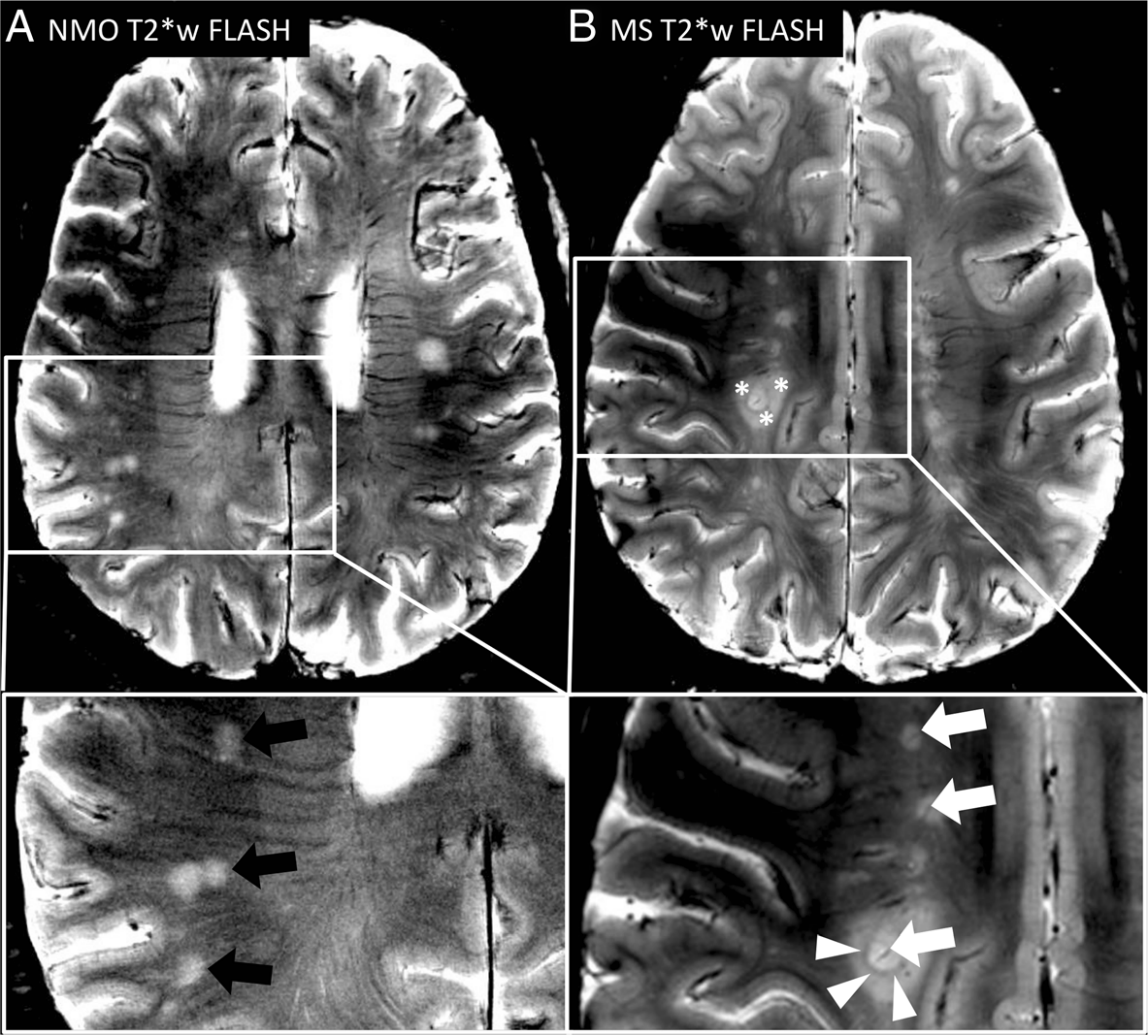
Neuromyelitis optica (NMO) versus multiple sclerosis (MS) lesion morphology. 7 T T2*w FLASH images from one exemplary NMO (**a**) and MS (**b**) patient are displayed. A small central vein can be displayed within the inner third of many MS lesions (*white arrows*). One acute MS lesion is characterised by a hypointense rim (*white arrowheads*) and surrounding edema (*asterisks*). Contrarily, a central vessel is not visible in NMO lesions (*black arrows*) despite using a very high spatial resolution of 0.2 mm × 0.2 mm × 2 mm

Neuromyelitis optica (NMO) is a potentially severe and disabling disease affecting primarily the spinal cord and the optic tracts [111]. Since the discovery of a pathogenic serum antibody against the astrocytic water channel aquaporin-4, it is no longer considered a variant of multiple sclerosis, but rather a disease entity of its own [112–122]. Distinct treatment regimens have been established in NMO, and drugs that are beneficial in MS might be harmful in NMO [123–128]. The distinction between NMO and MS, however, still remains puzzling in current clinical practice since brain white matter lesions—a hallmark of MS—are also detectable in more than 60 % of NMO cases during the course of the disease and a subset of NMO patients exhibit short cord lesions [129–132]. NMO and MS lesions can be described in more detail in high-resolution 7 T MR images. As stated above, MS lesions are characteristically centered by a small vein that is easily depictable at 7 T gradient echo images [71, 74, 82, 102]. Recently, two independent studies—each of them included ten patients with NMO spectrum disorders—described NMO lesion morphology at 7 T [92, 93]. Firstly, brain lesions were common in NMO as expected (92 lesions [93], 140 lesions [92]). A distinct central vein, however, was not commonly observed within NMO lesions: Kister and colleagues observed a central vein within 9 % (eight lesions) of all NMO lesions [93] and Sinnecker et al. detected an intra-lesional vein that was rarely centred within the lesion in 35 % (*n* = 49) of all NMO lesions (Fig. 4) [92]. In addition, T2*w hypointense rim-like alterations that can be often observed at the edge of MS plaques were only very rarely detectable around NMO lesions (*n* = 3) [92]. None of the two 7 T studies on NMO reported any cortical gray matter lesions in NMO patients [92, 93].

In summary, these 7 T MRI imaging characteristics may be used in the future to improve the differentiation between NMO and MS, which is highly relevant for the individual patient since therapeutic approaches in MS and NMO differ considerably [123–126]. The central vein sign is a potential future biomarker to distinguish MS from NMO patients. It is noteworthy that the sensitivity in detecting venous structures on 7 T gradient echo images largely relates to the imaging sequence, the post-processing, and the acquisition parameters such as the spatial resolution, flip angle, or echo time [102]. Thus, a “central vein cutoff value” for the differentiation of MS versus NMO lesions may vary in relationship to these parameters. An important limitation of current studies on NMO and 7 T MRI is the absence of spinal cord imaging at 7 T and small sample sizes [92, 93].

### Susac syndrome

**Fig. 5:**
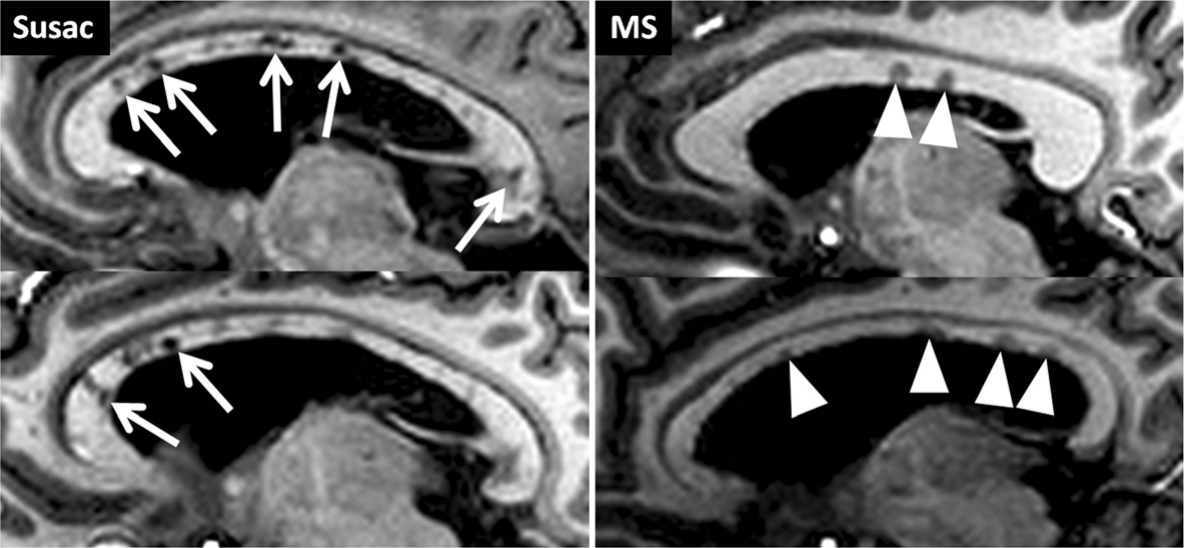
Callosal damage in Susac syndrome visualised on 7 T T1w images. The figure displays 7 T T1w MPRAGE images. Susac lesions (*white arrows*) are typically located within the centre of the corpus callosum and are often characterised by a prominent T1 hypointensity (*white arrows*) indicating severe tissue destruction. Contrarily, callosal MS lesions (*white arrowheads*) are often located adjacent to the ventricle within peripheral areas of the corpus callosum. These typically cap-shaped MS lesions are rather characterised by a moderate T1-hypointensity

Susac syndrome is an orphan disease that was first described by John Susac in 1979 as a clinical triad consisting of loss of vision, hearing loss, and encephalopathy that can present with headache or seizures [133]. It is considered a small vessel disease causing microinfarctions and damage to the cochlea, retina, and brain [133–140]. Susac syndrome is often a monophasic disease, but relapsing-remitting disease courses were described [135, 141]. In these cases, continuous immunosuppression may be beneficial, but larger systematic studies are not available to prove this assumption [142, 143]. Susac lesions within the corpus callosum can be imaged by MRI with a snowball-like or spike-like appearance [144]. Apart from callosal lesions, lesions are often detectable within the periventricular or deep white matter of Susac patients complicating the distinction from MS [95, 144]. A single study of five Susac and ten MS patients investigated the morphology of Susac lesions on 7 T MR images [95]. At 7 T, these lesions are rather unspecific in appearance without having a central vein or rim-like T2*w hypointense areas. In addition, callosal atrophy was detectable in many Susac patients presumably as a consequence of focal callosal damage and many cerebrospinal fluid (CSF) isointense black holes within the central part of the corpus callosum (Fig. 5). Contrarily, callosal MS lesions were often located in lateral areas of the corpus callosum showing less severe reduced T1w signal intensity values compared to Susac lesions. Future studies need to prove these initial findings in a larger sample size.

## Conclusions

An increasing number of 7 T MRI studies described unique features of MS lesions—most importantly, the central vein sign—that may be used in the future to differentiate MS lesions from brain lesions of other origin. Today there is, however, only limited evidence on these findings since many 7 T MRI studies comprise small patient cohorts or are hampered by a cross-sectional design. In addition, not all differential diagnoses of MS have been investigated at 7 T yet. From a more technical and practical perspective, technical limitations such as magnetic field inhomogeneity and economic as well as safety concerns have to be solved before widely applying 7 T in clinical practice. By then, we should aim to apply knowledge from these preliminary 7 T MRI studies to 3 T MRI platforms that are available for clinical imaging. Recently, different approaches to display venous structures within MS lesions at 3 T were published: FLAIR* combines FLAIR and T2*w images [145, 146], whereas susceptibility weighted FLAIR (sFLAIR) combines SWI and FLAIR images [102, 147]. In addition, optimised 3 T T2*w contrast may improve vessel detection at 3 T [148].

In the emerging field of personalised medicine, 7 T MRI may be used in patients with suspected neuroinflammatory disease such as MS, but conflicting clinical or paraclinical findings to support making the correct diagnosis early. Today, this should be done within the framework of clinical trials.

## Abbreviations

CIS: clinically isolated syndrome
CNS: central nervous system
CSF: cerebrospinal fluid
DIR: double inversion recovery
DNA: deoxyribonucleic acid
DSB: deoxyribonucleic acid double-strand breaks
FLAIR: fluid attenuated inversion recovery
FLASH: fast low angle shot
MPRAGE: magnetisation prepared rapid acquisition gradient echo
MP2RAGE: MPRAGE with multiple echoes
MRI: magnetic resonance imaging
MS: multiple sclerosis
NMO: neuromyelitis optica
OCT: optical coherence tomography
OR: optic radiation
RF: radiofrequency
sFLAIR: susceptibility weighted fluid attenuated inversion recovery
SNR: signal-to-noise ratio
T: Tesla
T1w: T1 weighted
T2*w: T2* weighted
T2w: T2 weighted
UHF: Ultrahigh field
VBM: voxel-based morphometry
